# Global integration of the hot-state brain network of appetite predicts short term weight loss in older adult

**DOI:** 10.3389/fnagi.2015.00070

**Published:** 2015-05-07

**Authors:** Brielle M. Paolini, Paul J. Laurienti, Sean L. Simpson, Jonathan H. Burdette, Robert G. Lyday, W. Jack Rejeski

**Affiliations:** ^1^Department of Radiology, Wake Forest University School of MedicineWinston-Salem, NC, USA; ^2^Translational Science Center, Wake Forest UniversityWinston-Salem, NC, USA; ^3^Department of Biostatistical Sciences, Wake Forest UniversityWinston-Salem, NC, USA; ^4^Department of Health and Exercise Science, Wake Forest UniversityWinston-Salem, NC, USA; ^5^Department of Geriatric Medicine, Wake Forest UniversityWinston-Salem, NC, USA

**Keywords:** weight loss, brain networks, graph theory, global efficiency, older adults, self regulation, anterior cingulate cortex (ACC)

## Abstract

Obesity is a public health crisis in North America. While lifestyle interventions for weight loss (WL) remain popular, the rate of success is highly variable. Clearly, self-regulation of eating behavior is a challenge and patterns of activity across the brain may be an important determinant of success. The current study prospectively examined whether integration across the Hot-State Brain Network of Appetite (HBN-A) predicts WL after 6-months of treatment in older adults. Our metric for network integration was global efficiency (GE). The present work is a sub-study (*n* = 56) of an ongoing randomized clinical trial involving WL. Imaging involved a baseline food-cue visualization functional MRI (fMRI) scan following an overnight fast. Using graph theory to build functional brain networks, we demonstrated that regions of the HBN-A (insula, anterior cingulate cortex (ACC), superior temporal pole (STP), amygdala and the parahippocampal gyrus) were highly integrated as evidenced by the results of a principal component analysis (PCA). After accounting for known correlates of WL (baseline weight, age, sex, and self-regulatory efficacy) and treatment condition, which together contributed 36.9% of the variance in WL, greater GE in the HBN-A was associated with an additional 19% of the variance. The ACC of the HBN-A was the primary driver of this effect, accounting for 14.5% of the variance in WL when entered in a stepwise regression following the covariates, *p* = 0.0001. The HBN-A is comprised of limbic regions important in the processing of emotions and visceral sensations and the ACC is key for translating such processing into behavioral consequences. The improved integration of these regions may enhance awareness of body and emotional states leading to more successful self-regulation and to greater WL. This is the first study among older adults to prospectively demonstrate that, following an overnight fast, GE of the HBN-A during a food visualization task is predictive of WL.

## Introduction

Obesity has become a public health crisis in North America, not sparing a quickly expanding population of older adults (Mathus-Vliegen et al., 2012; Ogden et al., 2014). Although lifestyle interventions remain the most popular treatment, there is considerable variability in weight loss (WL) during the intensive phase of treatment and, unfortunately, weight regain is common (Kramer et al., 1989; Wing and Hill, 2001; Rejeski et al., 2011). In the current study, we sought to examine whether functional brain network integration, as measured by global efficiency (GE) within five regions formerly described as the hot-state brain network of appetite (HBN-A; Paolini et al., 2014), was predictive of WL during the intensive phase of active treatment.

**Figure 1:**
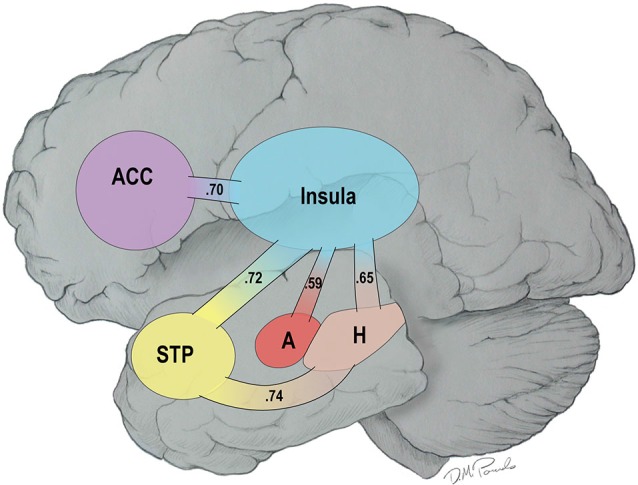
**Connections among the Hubs of the Hot-State Brain Network of Appetite in a Hot- vs. a Cold-State: Values in the Paths Represent Effect Sizes for Direct Connections**.

Recent reviews have noted that the primary cause of obesity—the overconsumption of food—is complex. Overconsumption is often due to both homeostatic dysregulation (bottom-up) and to dysfunctional central processing (top-down), the latter being referred to as the hedonic etiology of obesity (Lowe and Butryn, 2007; Stice et al., 2013). In line with the concept of homeostatic dysregulation, Loewenstein (2005) has proposed that individuals move into and out of “hot states” dynamically over the course of a day as a function of changes in affect or visceral cues making them vulnerable to dysfunctional health behaviors such as overeating. Recently, we demonstrated that resting-state brain networks are highly vulnerable to the “hot-state” of short term food restriction (Paolini et al., 2014). Specifically, fourteen older, overweight or obese adults visited our laboratory on two different occasions. On the first visit, they were fed a controlled breakfast, were not allowed to eat for 2.5 h, and then consumed a liquid meal replacement to create *a cold state* (low hunger). On a randomly assigned second visit, they consumed water after the 2.5 h of food restriction to create *a hot state* (increased feelings of hunger). All participants underwent resting functional MRI (fMRI) scans from which functional brain networks were constructed. The results of this study revealed that there was greater connectivity in a highly integrated network during the hot than cold state—the HBN-A. This network included five hubs: the insula, the anterior cingulate cortex (ACC), the superior temporal pole (STP), the amygdala and the hippocampus. In Figure 1, we use a cartoon to illustrate the greater number of statistically significant connections observed between the hubs of the HBN-A during a hot- vs. a cold-state (*p* < 0.05) (Paolini et al., 2014); the numbers reflect effect sizes.

After examining research which has shown that greater activity in reward centers of the brain is conducive to weight-regulation (Stice et al., 2010; Hege et al., 2013; Gearhardt et al., 2014; Wang et al., 2014), we hypothesized that it was the brain’s ability to integrate reward information globally that would be predictive of intentional WL. Within network theory, this makes GE a particularly relevant metric because it captures the potential for distributed processing or integration within and across a network (Latora and Marchiori, 2001; Rubinov and Sporns, 2010). Thus, in the current study, we examined whether GE of the HBN-A, following an overnight period of fasting, was related to WL during the first 6-months of intensive treatment among obese, older adults with cardiometabolic dysfunction. Our primary hypothesis was that after controlling for treatment conditions, baseline weight, sex, age, and change in self-regulatory efficacy (a measure of top-down control related to eating in high-risk contexts), higher baseline levels of GE across the HBN-A during the active visualization of food-cues would be significantly associated with 6-month WL.

## Methods

The present study involves ancillary imaging that is part of an ongoing randomized clinical trial involving WL, the Cooperative Lifestyle Intervention Program-II (CLIP-II) trial (Marsh et al., 2013). In short, CLIP-II randomized 252 obese, older adults with cardiovascular disease (CVD) or metabolic syndrome (MetS) into a WL only treatment, aerobic exercise training (AT) + WL, or resistance exercise training (RT) + WL for 18 months (Marsh et al., 2013). Since our focus was WL during the intensive phase of the interventions, we restricted our analysis to the first 6-months of therapy and statistically controlled for treatment in the linear model; we also cannot reveal treatment assignment prior to completion of the main study.

### Participants

Sixty-six participants who were enrolled during the first year of the CLIP-II study participated in this ancillary imaging project. The cohort and methods for this study have been well characterized (Marsh et al., 2013). Briefly, participants were either overweight or obese (BMI ≥ 28 kg/m^2^ but < 42 kg/m^2^) community dwelling men and women between the ages of 60 and 79 years with a documented history of CVD or an ATP III diagnosis of MetS. CVD included myocardial infarction (MI), percutaneous transluminal coronary angioplasty (PTCA), chronic stable angina, or cardiovascular surgery. Participants also had low levels of physical activity (<60 min of moderate intensity physical activity per week) and self-reported disability. The Weight Efficacy Lifestyle Questionnaire (WEL) was administered at both the baseline visit and the 6 month follow-up visit for the parent study (Clark et al., 1991).

After the participants had completed screening and baseline testing for the parent study, they were contacted via phone to discuss participation in and screening for the ancillary study. In addition to the inclusion/exclusion criteria for the parent study, participants were excluded for (1) visual acuity less than approximately 20/40 (corrected); (2) severe hearing loss; (3) claustrophobia; (4) MR-incompatibility (implant or other incompatible foreign object in the body); (5) depression treated by antidepressants unless on a stable regimen for more than 2 months; and (6) serious CNS trauma as defined by the history of acquired sub or epidural hematomas or loss of consciousness for greater than 5 min. Following successful completion of the phone screen, participants completed one 2.5 h experimental session at Wake Forest School of Medicine receiving $50 to compensate for their time. Informed consent was obtained on all participants and the study protocol was approved by the Wake Forest University School of Medicine Institutional Review Board.

Of the original 66 participants that agreed to participation, 2 participants withdrew during the MRI scan due to claustrophobia, 2 ended up being ineligible for the parent study, and 3 withdrew from the parent study during the first 6-months of treatment leaving a final *n* of 56.

### Measure of Self-Regulatory Efficacy

*Weight Efficiency Life-Style Questionnaire (WEL)*: The WEL, a 20-item measure developed by Clark et al. (1991), was employed to assess self-regulatory efficacy related to eating behavior. The measure has five subscales (negative emotions, food availability, social pressure, physical discomfort, and positive activities) but can be scored as a single measure as was done in the current study. Participants rated their confidence to resist the desire to eat using a 10-point scale ranging from 0 (not confident) to 9 (very confident). A total score was calculated by summing all items; therefore, scores range from 20 to 180.

### Experimental Protocol for the Scanning Visits

Once participants expressed interest in being involved in the imaging study and passed the telephone screening, an MRI appointment was arranged. They then completed a 2.5 h visit beginning in the early morning either around 7:15 a.m. or 9:15 a.m. and were asked to arrive in a fasting state, having not eaten breakfast or consumed anything other than water. Upon arrival, participants were led to a quiet, private room where informed consent was obtained by the study staff. The research staff then administered the MRI safety form and led the participant in a practice session of the tasks to be completed during the fMRI.

Two functional trials were completed during the MRI. The first trial was a 5-min resting state scan during which individuals relaxed with their eyes open viewing a cross on the rear projection screen. The second task was a 5-min food visualization task where participants viewed their four favorite food words on an MR-compatible rear projection screen; each word was presented for 30 s. Participants were instructed to visualize the food with all five senses for the entire time the food word was on the screen.

### Scanning Protocol

MRI data were obtained on a Siemens MEGNETOM SKYRA 3T scanner using a 32-channel head coil with a gradient strength equal to 45 mT/m at 200 T/m/s. The scanning protocol included anatomical imaging, one resting state fMRI and a food-cue fMRI task scan. Functional images for the network analyses measured the T2*-relaxation rate that is sensitive to blood oxygenation level dependent (BOLD) changes (Ogawa et al., 1990). As brain activity changes, the oxygen content of the blood in the same area also changes. Thus, the T2* signal is an indirect measure of changes in neural activity. All fMRI data was used to create brain networks for each individual in native space. High-resolution (1.0 × 1.0 × 1.0 mm) T1-weighted structural scans were acquired in the sagittal plane using a single-shot 3D MPRAGE GRAPPA2 sequence (Scan time = 5 min and 30 s, TR = 2.3 s, TE = 2.9 ms, TI = 900 ms, flip angle = 9°). Functional imaging or BOLD-weighted images (3.5 × 3.5 × 5.0 mm) were acquired using a single-shot echo-planar imaging sequence (Scan time = 5 min and 20 s, TR = 2.0 s, TE = 25 ms, flip angle = 75°). The scanning planes were oriented parallel to the anterior–posterior commissure line and extended from the superior extent of motor cortex to the base of the cerebellum.

### Imaging Processing and Network Analyses

Functional and structural data were preprocessed using SMP8.^1^ The first 20 s of the scan were discarded to allow for signal equilibration. Functional images were then realigned, slice-time corrected and co-registered to a skull-stripped version of the accompanying structural data. During image acquisition, the pre-scan normalize function was turned off for all 60 baseline scans. Pre-scan normalize is used to correct for the inherent bias in tissue signal based on location (i.e., tissue closer to the head coil has higher signal values than deeper brain structures). The built-in inhomogeneity correction function in SPM8 was utilized to remove this bias in the first volume of the functional data series. The parameters used on the first volume were then applied to the remainder of the functional data volumes images in the series.

Images were smoothed using a 2 voxel (8 × 8 × 8 mm) smoothing kernel, band-pass filtered (0.009–0.08 Hz) to limit physiological noise and low-frequency drift, and globally normalized to the image mean. Confounding signals were regressed out of the functional data and included 6 rigid-body transformation parameters generated during the realignment process and 3 mean signals (whole-brain, white matter, and cerebrospinal fluid (CSF)). The mean signals for the three tissue types were determined by masking the functional data with masks of the individual segmented tissue images generated with the unified segmentation in SPM8. Also, the functional data were motion corrected using a protocol designed to eliminate scan volumes with both excessive frame-wise displacement and BOLD signal change (Power et al., 2012). Networks were created and analyzed in native space to limit further data manipulation providing greater confidence in the time series signal.

For each individual, a correlation matrix was then created by computing Pearson correlations between all possible voxel pairs. Symmetrical matrices resulted, where each cell (ij) represented the correlation coefficient between nodes i and j. A threshold was then applied to the correlation matrix, and all cells that surpassed this threshold were assigned a value of 1 while remaining cells were assigned a value of zero. The size of each network ranged from 19,526 and 23,618 nodes based on the subject’s head size. The threshold was defined so that the relationship between the number of nodes and average number of connections at each node was consistent across subjects. This thresholding approach ensured comparable connection densities regardless of the number of network nodes. Specifically, the relationship *S* = log (*N*)/log(*K*) was the same across subjects, with *N* = number of nodes and *K* = average degree (Hayasaka and Laurienti, 2010). The threshold *S* = 2.5 was used for this paper. This resulted in connection densities meeting expected values based on network size (Laurienti et al., 2011). All remaining analyses were completed using the binary symmetrical adjacency matrixes.

GE (Latora and Marchiori, 2001) was used to assess the integrative capacity of the network and was calculated for each study participant at the nodal level. GE is the inverse of the average shortest path-length of the node and is defined as $GE=\frac{N}{\sum_{i}L}$ where *L* = shortest path-length from node (i) to every other network node and *N* = number of nodes in the network. Consequently, this metric ranges from 0 to 1. A node that is directly connected to all nodes in the network would have a GE of 1 whereas a disconnected node would have a GE of 0. Since GE incorporates the number of nodes in the network (N), the GE metric is readily comparable across networks of different sizes.

### Region-of-Interest (ROI) Analysis

To quantify GE for various ROIs, we warped the Automated Anatomical Labeling (AAL) atlas (Tzourio-Mazoyer et al., 2002) to each subject’s native-space structural brain image. First, the structural brain image was warped to standard space using the unified segmentation function in SPM8. The inverse warping parameters from the SPM were then applied to the AAL atlas for each participant. A nearest neighbor interpolation was used to ensure the boundaries of the atlas regions were respected (i.e., no voxel was included in more than one atlas region).

For our analysis, the following ROIs were used: insula, amygdala, STP, ACC, and parahippocampal gyrus, all defined by the AAL atlas. For each ROI both the left and right corresponding regions were included. The choice of these ROIs was based on our prior work with the HBN-A (Paolini et al., 2014); however, there were two exceptions. First, we used the entire ACC as defined by the AAL atlas for the present analysis; whereas, we used a sphere with radius of 10 mm previously. Second, we accessed the AAL’s region of the parahippocampal gyrus because this region mapped more closely onto the region of interest from our former work compared to the hippocampus (Tzourio-Mazoyer et al., 2002; Maldjian et al., 2003; Paolini et al., 2014). For each participant, the average GE was calculated for all the network nodes that fell within each of the 5 ROIs.

For the secondary analysis, ROIs were created for the default mode network (DMN). These ROIs were centered on coordinates previously defined in the literature (Shirer et al., 2012). The precuneus was a 12 mm sphere centered at 0, −50, 32 coordinates in MNI space. The left and right parietal regions were 16 mm spheres centered at −48, −60, 37 and 49, −64, 38 coordinates in MNI space, respectively. Finally, the medial prefrontal region was created by a 20 mm sphere centered at 0, 54, 4 in MNI space.

### Statistical Analyses

Means and standard deviations or medians and the 25th and 75th percentiles were used as measures of central tendency and variability. A principal components analysis (PCA) was applied to the GE data from the 5 regions of the HBN-A during the food visualization task to evaluate the integrity of this network. The primary data analytic technique was linear regression, regressing baseline weight, treatment assignment, age, sex, baseline adjusted change in self-regulatory efficacy across 6-months, and various measures of GE on 6-month WL. The variable inflation factor (VIF) was utilized to check for co-linearity. All analyses were conducted using SPSS version 22. Preliminary analyses were conducted to determine whether we should focus our attention on data from either the resting state scan or the food visualization task, and whether to consider using a factor score from the PCA of the 5 hubs of the HBN-A or the raw GE data from the 5 individual hubs.

## Results

The study sample consisted of 14 men and 42 women with 20 African Americans and 36 non-Hispanic Whites. Participants had a mean (SD) age of 67.55 (5.06) years with a Body Mass Index of 34.38 (3.69) kg/m^2^. The average Montreal Cognitive Assessment (MOCA) was 25.96 (2.39). The mean (SD) GE scores for the 5 regions of the HBN-A during the food visualization task were as follows: insula 0.135 (0.020), ACC 0.139 (0.020), amygdala 0.139 (0.024), STP 0.106 (0.019), and the parahippocampal gyrus 0.128 (0.024). These GE metrics for the 5 hubs of the HBN-A were highly integrated and constituted a single underlying dimension of function as evidenced by examination of the scree plot from a Principal Component Analysis (PCA). The eigenvalue for this single dimension was 3.82, accounting for 76.56% of the variance in these GE scores. The next largest eigenvalue was only 0.456, reinforcing the highly integrated nature of the 5 regions. Also, the correlations of the 5 regions with the principal component were all large: 0.90 for the insula, 0.87 for the amygdala, 0.85 for the STP, 0.85 for the ACC, and 0.90 for the parahippocampal gyrus. Across the 6-months, the mean (SD) WL was 17.77 (10.49) pounds; this variable was normally distributed as confirmed by visual inspection of a histogram and a non-significant Kolmogorov-Smirnov test (*p* = 0.200).

### Preliminary Data Analyses

To examine whether our analyses should focus on responses during the resting state or food imaging task, we created a stepwise model in which we entered the GE of the ACC from rest followed by GE of the ACC from the food visualization task after controlling for planned covariates. We chose the GE of the ACC because this component of the HBN-A had the strongest relationship to WL of all 5 hubs (see secondary analyses below). The results of this stepwise analysis clearly favored the food visualization task in that, although the ACC during the resting state accounted for 12.7% of the variance in WL beyond the covariates (*p* = 0.011), entry of the ACC from the food visualization task added another 4.9% of the variance (*p* = 0.03). In order to decide whether we would focus on the factor score from the PCA or the ACC of the HBN-A, we constructed a stepwise model entering the factor score during the food visualization task followed by the GE of the ACC during this task. Interestingly, the raw GE data from this specific hub of the HBN-A added another 7.1% of variance in explaining WL over and above the factor score and covariates (*p* = 0.010). Thus, in subsequent analyses, we focus exclusively on data collected during the food visualization task and the specific hubs of the HBN-A as opposed to factor scores.

### Evaluation of the Primary Hypothesis

As shown in model 1 of Table 1, the combined effects of the covariates in a linear regression accounted for a statistically and clinically meaningful percent of the variance in WL—36.9%. Examination of the corresponding *p*-values and standardized regression coefficients for each covariate provided in our final model found in Table 2 reveals that, after controlling for the effect of treatment, the amount of weight lost during the intensive phase of the intervention was greater for (a) men than women, (b) older than younger participants, (c) individuals who were heavier at baseline, and (d) those who experienced increased self-efficacy for control over eating in challenging situations.

**Table 1:** Models for R^2^ explained by covariates and R^2^ change with the addition of various regions of interest*.

Model	Change in *R*^2^	*F* for Change	*df*	*p*-Value
#1. Covariates	36.9%	4.77	6.49	0.001
#2. *GE for ACC* added to Covariates	14.5%	14.34	1.48	0.000
#3. *All 5 Hubs of the HBN-A* added to the Covariates	19.0%	3.80	5.44	0.006
#4. GE of ACC during Rest State added to model 2	3.1%	3.16	1.47	0.08
#5. *GE for Total Brain* added to model 2	0.2%	0.16	1.47	0.69
#6. *DFN* added to model 2	5.1%	1.28	4.44	0.29

*Note. Models 4–6 represent alternative predictors models within the same step.

**Table 2:** **Results of linear regression model on change in weight (lbs., 6 month—baseline): covariates + ACC added with stepwise procedure**.

Effect	Unstandardized Coefficients	Standardized Coefficients	*t*	*p*-value
Intercept	−27.69
Treatment* Vector 1	−8.74	−0.42	−3.25	0.002
Vector 2	−2.81	−0.12	−0.929	0.357
Baseline Weight	−0.165	−0.45	−3.80	0.000
Sex	11.59	0.48	3.66	0.001
Age in Years	−0.645	−0.31	−2.55	0.014
Self-Efficacy	−0.13	−0.39	−3.72	0.001
ACC: Global Efficiency	−195.42	−0.42	−3.79	0.000

*Dummy coding was used to control for the 3 treatments, thus requiring 2 vectors. Because treatment was used as a covariate and we did not want to reveal treatment assignment prior to completion of the main study, we do not discuss the treatment effect any further. The continuous predictor variables were centered to facilitate interpretation of the intercept.

**Figure 2:**
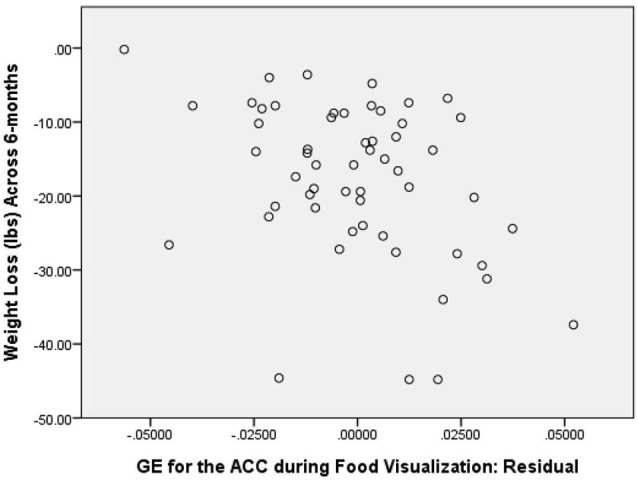
**Correlation between GE of the ACC during Food Visualization, Residualized for Covariates, and 6-Month Weight Loss**.

As a first step in evaluating the primary study hypothesis, we added the 5 hubs of the HBN-A to the block of covariates using a stepwise regression procedure to identify whether there was a single hub that was driving the effect that the HBN-A had on WL. In other words, after controlling for the covariates, we allowed the computer program to identify which hub or group of hubs added unique variance to explaining WL over and above the covariates. Because the hubs of the HBN-A are so highly integrated, as shown by the results of the PCA, we expected that only a single hub would be selected. Model 2 in Table 1 reveals that GE for the ACC proved to be this hub, accounting for an additional 14.5% of the variance in WL. Also, as shown by examination of the standardized regression coefficients in Table 2, the ACC was important relative to other variables in the model. This 14.5% of explained variance that the ACC contributed to WL over and above the covariates was an increase over the simply bivariate common variance between the ACC and WL of 8%, *p* = 0.03; a plot of the residualized values for GE of the ACC vs. WL can be found in Figure 2. Not surprisingly, in this stepwise entry of the HBN-A into the model, other than the ACC, the only hub that approached statistical significance was the insula with a *p*-value = 0.06; the remaining 3 hubs had *p-values* ranging from 0.25 and 0.66. However, because the HBN-A is a highly-integrated network, as a second step, we created a model that forced in all 5 hubs of the HBN-A after entry of the covariates. As shown in model 3 of Table 2, this resulted in a change in the R^2^ beyond the covariates of 19% as compared to 14.5% with only GE of the ACC in the model. Thus, our full models, combining information from the known correlates of WL with either the GE of the ACC or the GE of all 5 hubs of the HBN-A assessed during the active visualization of food explained over 50% of the variance in WL, 51.4% and 55.9%, respectively.

To confirm that the GE of the ACC was in fact the single most important hub of the HBN-A, we conducted separate regression models for each hub of the HBN-A, entering each hub individually after the covariates. The partial correlations for each hub in these models with WL were as follows: −0.38 for the ACC, −0.27 for the hippocampus, −0.20 for the insula, −0.22 for the amygdala, and −0.29 for the STP. The ACC accounted for 6.03% more variance in WL than any other single hub of the HBN-A.

### Secondary Analyses

As secondary analyses, we evaluated whether the GE for the resting state ACC, the GE for the total brain during the food visualization task, or the GE for the four regions of the DMN explained variance in WL beyond that accounted for by the covariates and the ACC of the HBN-A during the visualization of food. This aim was achieved by examining change in R^2^ that occurred with their addition to the model shown in Table 2. As shown in models 4, 5 and 6 of Table 1, neither the GE for the ACC during the resting state, the GE associated with the total brain, nor the GEs for the four hubs of the DMN explained additional variance in WL beyond what was achieved with our final model which included the covariates and the GE of the ACC during the food cue visualization task. We also ran stepwise models in which either the GE from the ACC at rest, the GE for the total brain during the visualization of food, or the DMN was forced into each model prior to the stepwise entry of the GE for the ACC during the visualization of food. In each case, the GE for the ACC during the food visualization task added significant variance to each model: 4.9%, *p* = 0.030 for the GE of the ACC during rest; 5.3%, *p* = 0.028 for GE of the total brain; and 5.7%, *p* = 0.021 for the DMN. Finally, we explored whether there were any meaningful interaction terms between GE of the ACC and the covariates of interest. None of these interactions proved to be significant (*p*-values > 0.05) including interactions with treatment.

## Discussion

Using PCA, and consistent with our previous work (Paolini et al., 2014), we were able to demonstrate that the five regions of the HBN-A were highly interrelated during a food visualization task that followed an overnight period of food restriction. We want to emphasize that the HBN-A was originally conceptualized in line with a recent review by Stice and colleagues and recognizing the inherent limitations of fMRI (Stice et al., 2013). The study population involved obese, older adults who had cardiometabolic dysfunction and were participating in a community-based WL trial. Consistent with our study hypothesis, we found that baseline GE of the HBN-A during a food cue visualization task accounted for 19% of the variance in WL following 6-months of active treatment. This effect was in addition to the 36.9% of the variance due to treatment assignment and several known correlates of WL including initial body weight, sex, age, and change in self-efficacy related to control of eating behavior. Given the additional variance that the HBN-A accounted for over and above self-efficacy, an intriguing hypothesis is that self-efficacy reflects a conscious component of self-regulation (top-down), whereas the HBN-A is capturing an unconscious, bottom-up self-regulatory influence on WL. Interesting, in a stepwise regression procedure, we found that the primary driver of the HBN-A was the ACC, accounting for 14.5% of the overall 19% of the variance captured by this network. Interpretation of the standardized regression coefficient for the GE of the ACC (−0.42) indicates that, after controlling for covariates in the model, a change of 1 standard deviation unit in the GE of the ACC is associated with 0.42 further decrease of a standard deviation unit in WL—a moderate effect. At the same time, as evident from results of the PCA and previous work by our group (Paolini et al., 2014), we want to emphasize that the ACC is highly integrated with the other hubs of the HBN-A. Also, from a methodological perspective, network theory argues for the importance of connectivity across the brain as opposed to region-specific explanations for behavior (Pessoa, 2014). That being said, a single network component can have more or less influence on the system behavior than other network components. We suggest that in the current study, the ACC was a key driver of system behavior, but that this influence only occurs because the ACC is a member of the HBN-A.

Secondary analyses were then conducted to evaluate whether GE metrics for the resting state HBN-A, the overall brain, or the DMN explained variance in WL when added to the parsimonious model described in Table 2; that is, a model including the combined effects of the covariates and the ACC of the HBN-A which together accounted for 51.4% of the variance in WL. None of these variables exceeded the criterion for entry into the model. Furthermore, we checked to determine what would happen if the GE of the ACC was not forced in the model prior to the competing GE metrics. In each case, the GE of the ACC always was selected for entry after the covariates as oppose to the GE of the resting state, total brain, or DMN. This adds confidence to our position that the HBN-A is an important network in understanding WL among older, obese adults with cardiometabolic dysfunction.

The obvious question that needs to be considered is why is this brain signature prospectively related to WL? As a caveat, we would like to begin by emphasizing that this is a single study that was not mechanistic by design. As research on this topic continues, it is important to emphasize that the regions of the HBN-A are all limbic or paralimbic and are important for processing visceral sensations and for the related emotional elaboration of these sensations (Paolini et al., 2014). Of interest is the fact that, in the stepwise model selection of the five regions of the HBN-A, the only other hub besides the ACC that approached statistical significance was the insula with a *p*-value = 0.06. In research by other investigators, the insula has been found to work in concert with the ACC to translate visceral and emotional processing into behavioral consequences and has a long history of being implicated in eating behavior (Critchley, 2004; Rothemund et al., 2007; Kringelbach et al., 2012; Uddin et al., 2014). Historically, the ACC has been associated with goal-directed behavior and attention regulation for both cognitive (dorsal portion) and emotional processing (ventral portion) (Devinsky et al., 1995; Bush et al., 2000; Mohanty et al., 2007; Gasquoine, 2013). Generally speaking, traditional activation studies have shown that greater activity in the ACC is associated with obesity (Stoeckel et al., 2008; Gearhardt et al., 2014; Jensen and Kirwan, 2015), weight gain (Mathews et al., 2012), and food addiction (Gearhardt et al., 2011). Thus, the central role played by the ACC within the HBN-A is not surprising.

We want to emphasize that research in this areas by other investigators has relied on traditional activation methods, which identify isolated brain regions that become differentially active between tasks. The present approach used graph-theory to study the entire brain focusing on overall patterns of connectivity. Specifically, we used GE, a choice metric for capturing a region’s integration or reach throughout the entire brain network (Rubinov and Sporns, 2010); we found that WL was directly related to the greater reach of the ACC throughout the brain network. While it appears highly likely that important food-related processing does occur in the ACC (Stoeckel et al., 2008; Gearhardt et al., 2011, 2014; Mathews et al., 2012; Jensen and Kirwan, 2015), we hypothesize that it is the ACC’s role within the HBN-A and its ability to globally distribute this information throughout the brain that is most important for success with intentional WL. Integration may allow the HBN-A’s visceral and emotional information to be shared across the brain and to reach higher, cortical areas, potentially allowing unconscious information to enter into conscious awareness. Global sharing may provide individuals with superior body and emotional awareness and improve processing capabilities that foster adaptive self-regulatory behaviors; however, confirmation of this hypothesis awaits further controlled study. In previous work we found that, following recovery from a food challenge (i.e., visualization of food cues), individuals high in trait mindfulness had greater GE in the insula relative to individuals lower on trait mindfulness (Paolini et al., 2012). These individuals also have greater perceived self-control related to eating behavior (Paolini et al., 2012). Collectively, these data support the adaptive nature of maintaining distributed processing throughout the brain when exposed to food-related challenges and substantiate the work of Cozolino (2010) and Siegel (2007) who suggest that both horizontal and vertical brain integration is required for effective self-regulation.

It is important to mention three studies in the literature that have examined baseline brain activity and subsequent success or failure during an active WL intervention (Kishinevsky et al., 2012; Murdaugh et al., 2012; Hege et al., 2013; Weygandt et al., 2013), although this body of evidence has not employed state-of-the-art methods in network science. Weygandt et al. (2013) using fMRI found that higher activity in two regions of the frontal cortex, the VMPFC and DLPFC, during a food-related task requiring executive function predicted subsequent WL in a twelve-week low calorie dietary intervention (Weygandt et al., 2013). The other two studies identified regions outside of the frontal cortex. Murdaugh and colleagues (Murdaugh et al., 2012) found that greater baseline ACC activation in obese individuals was associated with less WL. Alternatively, Hege et al. (2013) reported an opposite pattern with magnetoencephalography (MEG). Specifically, they found that those individuals who were *successful* with WL had *greater* baseline brain activation in right temporal areas, including the hippocampus and the fusiform gyrus during a one-back visual memory task with food cues than individuals who struggled with WL. One likely reason that these studies may be in conflict is because they used different food-cue tasks and, therefore, were dynamically recruiting differing brain regions.

The current study is not without limitations. Although we feel that GE is an ideal metric for capturing the experience of hunger and, consequently, self-regulation during WL, it would have been ideal to replicate the findings of our original study on the HBN-A circuit using degree (Paolini et al., 2014). We were unable to do so for two reasons. First, as we demonstrated in that original work, degree appears to be associated with the state of being fed or unfed (i.e., regions of the HBN-A have higher degree in the unfed vs. the fed condition). Unfortunately, the current study was not designed to replicate this finding, as we did not have a fed condition. Second, the present network analyses were performed in native space; thus, each network had a different number of nodes and connections making it impossible to use the same analysis employed in the original study. In addition, we are unable to determine if our findings were specific to the “hot-state” created by food restriction. Since our participants fasted overnight, based on findings from our previous work (Paolini et al., 2014), we assumed that these individuals were experiencing a “hot-state.” However, without a cold/fed condition, we do not have the data to make this definitive conclusion. Future work should compare baseline brain network integration between a hot (i.e., fasting) and a cold (i.e., fed) condition.

In summary, after controlling for important covariates, this study demonstrated that distributed connectivity of the ACC and the entire HBN-A during food-cue visualization are important determinants of WL in obese, older adults with cardiometabolic dysfunction. The HBN-A is comprised of limbic and paralimbic regions important in processing visceral cues and emotions, and the ACC is a significant structure for translation of this awareness into behavioral consequences. Further research is warranted to examine whether a targeted intervention can improve brain integration across the regions of the HBN-A in those who have compromised function and whether these changes augment the success of WL therapy. In addition, attention needs to be directed to long-term WL and to the problem of weight regain that commonly follows intentional WL.

## Conflict of Interest Statement

The authors declare that the research was conducted in the absence of any commercial or financial relationships that could be construed as a potential conflict of interest.

## Footnotes

^1^www.fil.ion.ucl.ac.uk/spm/

## References

[B1] Bush G. Luu P. Posner M. I. (2000). Cognitive and emotional influences in anterior cingulate cortex. Trends Cogn. Sci. 4, 215–222. 10.1016/s1364-6613(00)01483-2 10827444

[B2] Clark M. M. Abrams D. B. Niaura R. S. Eaton C. A. Rossi J. S. (1991). Self-efficacy in weight management. J. Consult. Clin. Psychol. 59, 739–744. 10.1037/0022-006X.59.5.739 1955608

[B3] Cozolino L. J. (2010). The Neuroscience of Psychotherapy: Healing the Social Brain. New York: W. W. Norton and Company.

[B4] Critchley H. D. (2004). The human cortex responds to an interoceptive challenge. Proc. Natl. Acad. Sci. U S A 101, 6333–6334. 10.1073/pnas.0401510101 15096592 PMC404044

[B5] Devinsky O. Morrell M. J. Vogt B. A. (1995). Contributions of anterior cingulate cortex to behaviour. Brain 118(Pt. 1), 279–306. 10.1093/brain/118.1.279 7895011

[B6] Gasquoine P. G. (2013). Localization of function in anterior cingulate cortex: from psychosurgery to functional neuroimaging. Neurosci. Biobehav. Rev. 37, 340–348. 10.1016/j.neubiorev.2013.01.002 23313645

[B7] Gearhardt A. N. Yokum S. Orr P. T. Stice E. Corbin W. R. Brownell K. D. (2011). Neural correlates of food addiction. Arch. Gen. Psychiatry 68, 808–816. 10.1001/archgenpsychiatry.2011.32 21464344 PMC3980851

[B8] Gearhardt A. N. Yokum S. Stice E. Harris J. L. Brownell K. D. (2014). Relation of obesity to neural activation in response to food commercials. Soc. Cogn. Affect. Neurosci. 9, 932–938. 10.1093/scan/nst059 23576811 PMC4090951

[B9] Hayasaka S. Laurienti P. J. (2010). Comparison of characteristics between region-and voxel-based network analyses in resting-state fMRI data. Neuroimage 50, 499–508. 10.1016/j.neuroimage.2009.12.051 20026219 PMC2824075

[B10] Hege M. A. Stingl K. T. Ketterer C. Häring H. U. Heni M. Fritsche A.. (2013). Working memory-related brain activity is associated with outcome of lifestyle intervention. Obesity (Silver Spring) 21, 2488–2494. 10.1002/oby.20442 23512974

[B11] Jensen C. D. Kirwan C. B. (2015). Functional brain response to food images in successful adolescent weight losers compared with normal-weight and overweight controls. Obesity (Silver Spring) 23, 630–636. 10.1002/oby.21004 25645425

[B12] Kishinevsky F. I. Cox J. E. Murdaugh D. L. Stoeckel L. E. Cook E. W. 3rd Weller R. E. (2012). fMRI reactivity on a delay discounting task predicts weight gain in obese women. Appetite 58, 582–592. 10.1016/j.appet.2011.11.029 22166676

[B13] Kramer F. M. Jeffery R. W. Forster J. L. Snell M. K. (1989). Long-term follow-up of behavioral treatment for obesity: patterns of weight regain among men and women. Int. J. Obes. 13, 123–136. 2663745

[B14] Kringelbach M. L. Stein A. van Hartevelt T. J. (2012). The functional human neuroanatomy of food pleasure cycles. Physiol. Behav. 106, 307–316. 10.1016/j.physbeh.2012.03.023 22487544

[B15] Latora V. Marchiori M. (2001). Efficient behavior of small-world networks. Phys. Rev. Lett. 87:198701. 10.1103/physrevlett.87.198701 11690461

[B16] Laurienti P. J. Joyce K. E. Telesford Q. K. Burdette J. H. Hayasaka S. (2011). Universal fractal scaling of self-organized networks. Physica A 390, 3608–3613. 10.1016/j.physa.2011.05.011 21808445 PMC3146350

[B17] Loewenstein G. (2005). Hot-cold empathy gaps and medical decision making. Health Psychol. 24, S49–S56. 10.1037/0278-6133.24.4.s49 16045419

[B18] Lowe M. R. Butryn M. L. (2007). Hedonic hunger: a new dimension of appetite? Physiol. Behav. 91, 432–439. 10.1016/j.physbeh.2007.04.006 17531274

[B19] Maldjian J. A. Laurienti P. J. Kraft R. A. Burdette J. H. (2003). An automated method for neuroanatomic and cytoarchitectonic atlas-based interrogation of fMRI data sets. Neuroimage 19, 1233–1239. 10.1016/s1053-8119(03)00169-1 12880848

[B20] Marsh A. P. Janssen J. A. Ambrosius W. T. Burdette J. H. Gaukstern J. E. Morgan A. R.. (2013). The Cooperative Lifestyle Intervention Program-II (CLIP-II): design and methods. Contemp. Clin. Trials 36, 382–393. 10.1016/j.cct.2013.08.006 23974035 PMC3843993

[B21] Mathews J. Newcomer J. W. Mathews J. R. Fales C. L. Pierce K. J. Akers B. K.. (2012). Neural correlates of weight gain with olanzapine. Arch. Gen. Psychiatry 69, 1226–1237. 10.1001/archgenpsychiatry.2012.934 22868896

[B22] Mathus-Vliegen E. M. Basdevant A. Finer N. Hainer V. Hauner H. Micic D.. (2012). Prevalence, pathophysiology, health consequences and treatment options of obesity in the elderly: a guideline. Obes. Facts 5, 460–483. 10.1159/000341193 22797374

[B23] Mohanty A. Engels A. S. Herrington J. D. Heller W. Ho M. H. Banich M. T.. (2007). Differential engagement of anterior cingulate cortex subdivisions for cognitive and emotional function. Psychophysiology 44, 343–351. 10.1111/j.1469-8986.2007.00515.x 17433093

[B24] Murdaugh D. L. Cox J. E. Cook E. W. 3rd Weller R. E. (2012). fMRI reactivity to high-calorie food pictures predicts short- and long-term outcome in a weight-loss program. Neuroimage 59, 2709–2721. 10.1016/j.neuroimage.2011.10.071 22332246 PMC3287079

[B25] Ogawa S. Lee T. M. Kay A. R. Tank D. W. (1990). Brain magnetic resonance imaging with contrast dependent on blood oxygenation. Proc. Natl. Acad. Sci. U S A 87, 9868–9872. 10.1073/pnas.87.24.9868 2124706 PMC55275

[B26] Ogden C. L. Carroll M. D. Kit B. K. Flegal K. M. (2014). Prevalence of childhood and adult obesity in the United States, 2011–2012. JAMA 311, 806–814. 10.1001/jama.2014.732 24570244 PMC4770258

[B27] Paolini B. Burdette J. H. Laurienti P. J. Morgan A. R. Williamson D. A. Rejeski W. J. (2012). Coping with brief periods of food restriction: mindfulness matters. Front. Aging Neurosci. 4:13. 10.3389/fnagi.2012.00013 22685430 PMC3368241

[B28] Paolini B. M. Laurienti P. J. Norris J. Rejeski W. J. (2014). Meal replacement: calming the hot-state brain network of appetite. Front. Psychol. 5:249. 10.3389/fpsyg.2014.00249 24723901 PMC3971177

[B29] Pessoa L. (2014). Understanding brain networks and brain organization. Phys. Life Rev. 11, 400–435. 10.1016/j.plrev.2014.03.005 24819881 PMC4157099

[B30] Power J. D. Barnes K. A. Snyder A. Z. Schlaggar B. L. Petersen S. E. (2012). Spurious but systematic correlations in functional connectivity MRI networks arise from subject motion. Neuroimage 59, 2142–2154. 10.1016/j.neuroimage.2011.10.018 22019881 PMC3254728

[B31] Rejeski W. J. Mihalko S. L. Ambrosius W. T. Bearon L. B. McClelland J. W. (2011). Weight loss and self-regulatory eating efficacy in older adults: the cooperative lifestyle intervention program. J. Gerontol. B Psychol. Sci. Soc. Sci. 66, 279–286. 10.1093/geronb/gbq104 21292809 PMC3078758

[B32] Rothemund Y. Preuschhof C. Bohner G. Bauknecht H. C. Klingebiel R. Flor H.. (2007). Differential activation of the dorsal striatum by high-calorie visual food stimuli in obese individuals. Neuroimage 37, 410–421. 10.1016/j.neuroimage.2007.05.008 17566768

[B33] Rubinov M. Sporns O. (2010). Complex network measures of brain connectivity: uses and interpretations. Neuroimage 52, 1059–1069. 10.1016/j.neuroimage.2009.10.003 19819337

[B34] Shirer W. R. Ryali S. Rykhlevskaia E. Menon V. Greicius M. D. (2012). Decoding subject-driven cognitive states with whole-brain connectivity patterns. Cereb. Cortex 22, 158–165. 10.1093/cercor/bhr099 21616982 PMC3236795

[B35] Siegel D. J. (2007). The Mindful Brain. New York: W. W. Norton and Company.

[B36] Stice E. Figlewicz D. P. Gosnell B. A. Levine A. S. Pratt W. E. (2013). The contribution of brain reward circuits to the obesity epidemic. Neurosci. Biobehav. Rev. 37, 2047–2058. 10.1016/j.neubiorev.2012.12.001 23237885 PMC3604128

[B37] Stice E. Yokum S. Bohon C. Marti N. Smolen A. (2010). Reward circuitry responsivity to food predicts future increases in body mass: moderating effects of DRD2 and DRD4. Neuroimage 50, 1618–1625. 10.1016/j.neuroimage.2010.01.081 20116437 PMC3987805

[B38] Stoeckel L. E. Weller R. E. Cook E. W. 3rd Twieg D. B. Knowlton R. C. Cox J. E. (2008). Widespread reward-system activation in obese women in response to pictures of high-calorie foods. Neuroimage 41, 636–647. 10.1016/j.neuroimage.2008.02.031 18413289

[B39] Tzourio-Mazoyer N. Landeau B. Papathanassiou D. Crivello F. Etard O. Delcroix N.. (2002). Automated anatomical labeling of activations in SPM using a macroscopic anatomical parcellation of the MNI MRI single-subject brain. Neuroimage 15, 273–289. 10.1006/nimg.2001.0978 11771995

[B40] Uddin L. Q. Kinnison J. Pessoa L. Anderson M. L. (2014). Beyond the tripartite cognition-emotion-interoception model of the human insular cortex. J. Cogn. Neurosci. 26, 16–27. 10.1162/jocn_a_00462 23937691 PMC4074004

[B41] Wang G. J. Tomasi D. Volkow N. D. Wang R. Telang F. Caparelli E. C.. (2014). Effect of combined naltrexone and bupropion therapy on the brain’s reactivity to food cues. Int. J. Obes. (Lond) 38, 682–688. 10.1038/ijo.2013.145 23924756 PMC4010969

[B42] Weygandt M. Mai K. Dommes E. Leupelt V. Hackmack K. Kahnt T.. (2013). The role of neural impulse control mechanisms for dietary success in obesity. Neuroimage 83, 669–678. 10.1016/j.neuroimage.2013.07.028 23867558

[B43] Wing R. R. Hill J. O. (2001). Successful weight loss maintenance. Annu. Rev. Nutr. 21, 323–341. 10.1146/annurev.nutr.21.1.323 11375440

